# 5-Methoxytryptophan Sensitizing Head and Neck Squamous Carcinoma Cell to Cisplatitn Through Inhibiting Signal Transducer and Activator of Transcription 3 (STAT3)

**DOI:** 10.3389/fonc.2022.834941

**Published:** 2022-07-22

**Authors:** Yu-Chieh Su, Chih-Chun Wang, Jui-Hsi Weng, Shyh-An Yeh, Po-Jen Chen, Tzer-Zen Hwang, Huang-Chi Chen

**Affiliations:** ^1^ School of Medicine, College of Medicine, I-Shou University, Kaohsiung, Taiwan; ^2^ Division of Hematology-Oncology, Department of Internal Medicine, E-Da Hospital, Kaohsiung, Taiwan; ^3^ Department of Otolaryngology, E-Da Hospital, Kaohsiung, Taiwan; ^4^ Yuh-Ing Junior College of Health Care and Management, Kaohsiung, Taiwan; ^5^ Department of Radiation Oncology, E-Da Hospital, Kaohsiung, Taiwan; ^6^ Department of Medical Imaging and Radiological Sciences, I-Shou University, Kaohsiung City, Taiwan; ^7^ Department of Medical Research, E-Da Hospital, Kaohsiung, Taiwan; ^8^ Department of Internal Medicine, Kaohsiung Municipal Siaogang Hospital, Kaohsiung, Taiwan; ^9^ Division of Pulmonary and Critical Care Medicine, Department of Internal Medicine, Kaohsiung Medical University Hospital, Kaohsiung, Taiwan

**Keywords:** head and neck squamous cell carcinoma, cisplatin, 5-methoxytryptophan, STAT3, 4NQO

## Abstract

Head and neck squamous cell carcinoma (HNSCC) is a common cancer of the oral cavity. Cisplatin (CDDP) is the ideal chemo-radiotherapy used for several tumor types, but resistance to the drug has become a major obstacle in treating patients with HNSCC. 5-methoxytryptophan (5-MTP), a 5-methoxyindole metabolite of tryptophan metabolism, reduces inflammation-mediated proliferation and metastasis. This study aimed to assess the anti-oral cancer activity of 5-MTP when used alone or in combination with CDDP. Results showed that CDDP dose dependently reduced the growth of SSC25 cells but not 5-MTP. The combination of CDDP and 5-MTP exerted additional inhibitory effect on the growth of SSC25 cells by attenuating the phosphorylation of STAT3. In the 4-nitroquinoline-1-oxide-induced oral cancer mouse model, 5-MTP sensitized the reduction effect of CDDP on tumorigenesis, which restricted the tongue tissue in hyperkeratotic lesion rather than squamous cell carcinoma. The combination of CDDP and 5-MTP may be a potent therapeutic strategy for HNSCC patients with radiotherapy.

## Introduction

Head and neck cancer (HNC) is the sixth most common cancer worldwide and severely impacts the life quality of patients (1), especially those with severe drug resistance and low treatment responsiveness. HNC is a tumor with heterogenous groups consisting of the mucosal surface of the nasal and oral cavities, hypopharynx, larynx, and oropharynx (2). Head and neck squamous cell carcinoma (HNSCC) is the most common HNC, accounting for up to 90% of these tumors (3). Approximately 25% of patients with HNSCC develop a second cancer within 5 years after diagnosis; the secondary tumor is the most common cause of treatment failure, leading to the low survival rate of these patients (4). The incidence rate of oral cancer in Taiwan is one of the highest in the world, comprising 8.0% of all new cancers and 6.3% of all cancer deaths (5). Hence, new therapeutic approaches or agents must be developed to improve the HNSCC treatment and the quality of life of patients.

Cisplatin (CDDP) is activated by the replacement of a chloride ligand with H_2_O in the cytoplasm. Activated CDDP binds to DNA, generating intra-strand DNA crosslinks, which lead to defective DNA metabolic processes, including DNA replication and transcription in cancer cells (6). CDDP is a common and effective chemotherapy treatment available for patients with metastatic and recurrent HNSCC (7). Furthermore, the radiation therapy for patients with HNSCC is potentialized by cisplatin treatment (8). However, intrinsic or acquired resistance causes the clinical failure of CDDP treatment, resulting in therapy discontinuation. Furthermore, CDDP administration to resistant cases provides no curative effect and can even increase the chance of disease progression and adverse side effects, such as ototoxicity and nephrotoxicity (9, 10).

5-methoxytryptophan (5-MTP) is a 5-methoxyindole metabolite of tryptophan metabolism. Unlike 5-methoxyindoles that participate in the control of circadian rhythm in the pineal gland and retina, 5-MTP suppresses inflammatory-induced cancer cell growth and migration (11). 5-MTP attenuates reactive oxygen species formation and the excessive immune cell infiltration through controlling the inflammation by blocking p38 and nuclear factor (NF)-κB activation (12, 13). The suppression effect of 5-MTP on cyclooxygenase (COX)-2 expression inhibits epithelial mesenchymal transition and cancer cell migration and metastasis (14). A murine A549 xenograft tumor model demonstrated that the intraperitoneal injection of 5-MTP significantly reduces tumor growth up to 50% when compared with the vehicle control (15). Furthermore, 5-MTP reduces the invasion and metastasis of oral cancer *in vitro* and vivo (16). However, the antioral cancer mechanism of 5-MTP remains unclear.

Signal transducer and activator of transcription 3 (STAT3) is a crucial molecule for tumor cell proliferation and survival (17). Furthermore, STAT3 is involved in tumor-related immunosuppression at many levels. In the tumor microenvironment, the constitutive STAT3 activation could be prolonged by STAT3-mediated factors, including vascular endothelial growth factor (VEGF) and interleukin-10 (IL-10), which generate immunosuppression in innate and adaptive immunity (18, 19). In the present study, we aim to investigate the inhibitory effect and related mechanism of the combination treatment of CDDP and 5-MTP in HNSCC cells and 4-nitroquinoline-1-oxide (4NQO)-induced oral tumor mouse model. We first evaluated the anticancer activity of CDDP and 5-MTP in SCC25 cells by determining the proliferation of the cancer cells. We further compared the anticancer effect on SCC25 cells in response to concurrent CDDP and 5-MTP or single compound treatment. The sensitization effect of HNSCC to CDDP by the usage of 5-MTP on cell proliferation was confirmed in the 4NQO-induced oral mouse model.

## Results

### *In Vitro* Validation of CDDP and 5-MTP in Oral Cancer Cell Line

**Figure 1:**
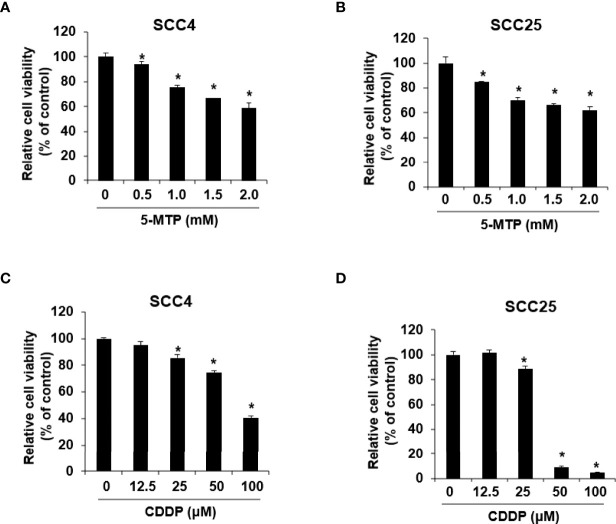
Reduction effect of Cisplatin (CDDP) and 5-methoxytryptophan (5-MTP) on the proliferation of SSC4 and SSC25 cells. 5-MTP reduced the proliferation of SCC4 **(A)** and SCC25 **(B)** cells. CDDP reduced the proliferation of SCC4 **(C)** and SCC25 **(D)** cells. Data are presented as the mean ± SD of at least three independent experiments. Asterisks indicate significant difference in drug-treated cells at indicated concentrations singly in comparison with DMSO-treated cells. *P < 0.05.

The anticancer activity of CDDP and 5-MTP on oral squamous cell carcinoma cells was evaluated by administering different doses of CDDP or 5-MTP to different cell lines, including SCC4 and SCC25, for 24 h. Cell viability was determined by cell viability assay. As shown in **Figure 1**, CDDP at 100 μM obviously reduced the proliferation of SCC4 cells, whereas CDDP at 50 and 100 μM obviously reduced the proliferation of SCC25 cells. 5-MTP at 0.5–2 mM obviously decreased the proliferation of SCC4 cells. The half maximal inhibitory concentration (IC_50_) of CDDP in SCC25 was 37.7 μM, whereas that in SCC4 was 99.1 μM. The IC_50_ values of 5-MTP in SCC4 and SCC25 were 2.7 and 3.1 mM, respectively. Collectively, these results showed that CDDP inhibited cancer cell growth in a dose-dependent manner and exhibited better reduction effect in SCC25 cells than in SCC4 cells. 5-MTP reduced the proliferation of cancer cells with a weak dose-dependent manner, indicating that 5-MTP may not be an ideal single agent for killing oral cancer cells.

### Combination Treatment of CDDP and 5-MTP in Oral Cancer Cells

**Figure 2:**
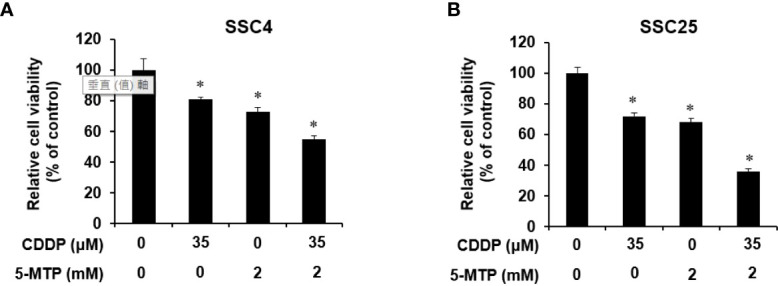
Additional inhibitory effect of the combination treatment of Cisplatin (CDDP) and 5-methoxytryptophan (5-MTP) on the proliferation of SCC4 and SCC25 cells. Combination treatment showed an additional inhibitory effect on the growth of SCC4 **(A)** and SCC25 **(B)** cells when compared with the single treatments. Data are presented as the mean ± SD of at least three independent experiments. Asterisks indicate significant difference in drug-treated cells at indicated concentrations singly in comparison with DMSO-treated cells. *P < 0.05.

Whether 5-MTP can increase the inhibitory effect of CDDP on oral cancer cell growth was examined. SCC4 and SCC25 cells were exposed to CDDP, 5-MTP, or both at indicated concentrations for 24 h, and then cell proliferation was examined. As shown in **Figure 2**, the combination treatment of CDDP and 5-MTP obviously reduced cancer cell growth compared with either treatment alone. This result suggested that the inhibitory effect of CDDP was sensitized by 5-MTP.

### Mechanism Validation of the Inhibitory Effect of CDDP and 5-MTP

**Figure 3:**
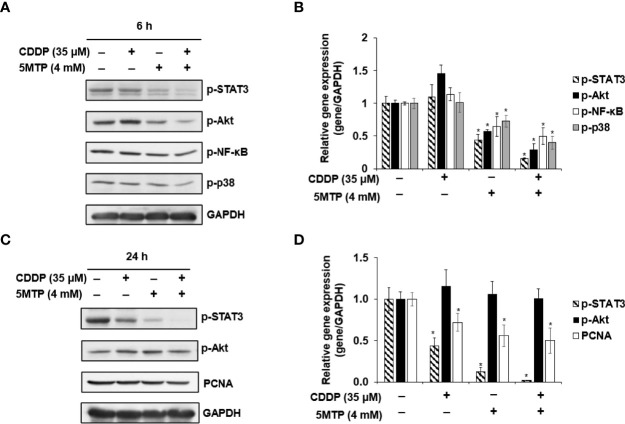
Inhibitory effect of the combination treatment of Cisplatin (CDDP) and 5-methoxytryptophan (5-MTP) on proliferation involved in the STAT3 signaling pathway. SCC4 and SCC25 cells were treated with CDDP, 5-MTP, or both at indicated concentrations for 6 h **(A, B)** or 24 h **(C, D)**. Total cell lysates were extracted at the indicated time points and subjected to western blot with relevant antibodies. GAPDH served as an equal loading control in western blot. Asterisks indicate significant difference in drug-treated cells at indicated concentrations singly in comparison with DMSO-treated cells. *P < 0.05.

The potential signaling pathways underlying the inhibitory effect of CDDP and 5-MTP on the growth of SCC25 cells were explored. The expression of proliferation signaling pathway-related proteins, including phospho-STAT3, phospho-NF-κB, phospho-Akt, and phospho-p38, was evaluated using western blot. The expression of proliferation cell nuclear antigen (PCNA) was obviously reduced in SCC25 cells after the combination treatment of CDDP and 5-MTP. SCC25 cells were treated with CDDP, 5-MTP, or both for 6 h to investigate further the mechanism by which the combination treatment reduces PCNA expression. The expression levels of phospho-NF-κB, phospho-Akt, and phospho-p38 were reduced in the CDDP and 5-MTP combination-treated cells (**Figures 3A, B**). Notably, the combination treatment of CDDP and 5-MTP obviously reduced the expression of phospho-STAT3 but not that of phospho-Akt at 24 h in SCC25 cells (**Figures 3C, D**).

### Validation of the Sensitization of Oral Cancer to CDDP by 5-MTP *In Vivo*

**Figure 4:**
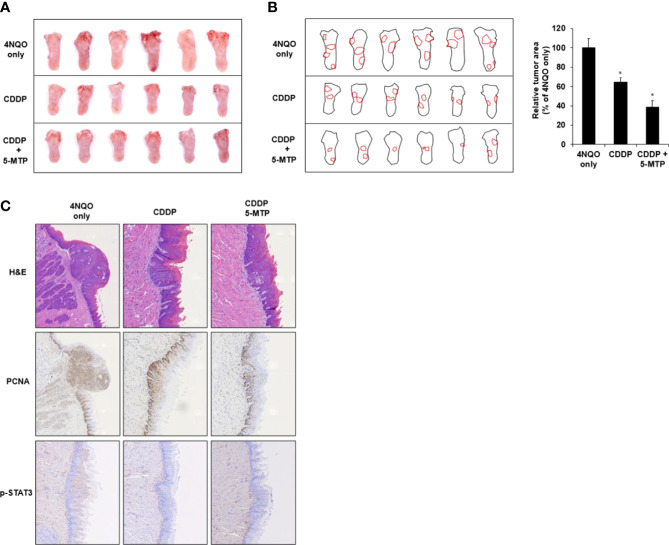
Additional inhibitory effect of the combination treatment of Cisplatin (CDDP) and 5-methoxytryptophan (5-MTP) in 4NQO-induced tongue oral squamous cell carcinoma (OSCC) mouse model. **(A)** Morphological observation of the DMSO- or drug-treated group in 4NQO-induced cancer mouse model. **(B)** Digital outlines of representative tongues of DMSO- or drug-treated mice. Asterisks indicate significant difference in drug-treated group singly in comparison with 4NQO-treated group. *P < 0.05 **(C)** hematoxylin–eosin or immunohistochemical staining with PCNA and p-STAT3 antibodies of tongue cancer with DMSO or drug treatment.

The effect of CDDP or CDDP combined with 5-MTP on oral cancer biogenesis was evaluated. Drinking water with 4NQO was provided to BALB/c mice for 18 weeks, and then the 4NQO water was reverted to normal water for another 4 weeks. After the tumor became visible on the tongue, the animals were administered with CDDP (2 mg/kg) and 5-MTP (35 mg/kg) twice a week for 4 weeks. The tumor burden in the treated groups decreased compared with that in the control group. Notably, the combination treatment group showed more obviously reduced tumor burden than the CDDP-treated group (**Figure 4A**). Tumor load was outlined as a digital image, and the relative tumor area of the CDDP or 5-MTP group was significantly reduced when compared with 4NQO only group (**Figure 4B**). In the histologic examination, the tongue of the control group showed that most of the tissue contained dysplasia and squamous cell carcinoma, and the CDDP-treated group showed less squamous cell carcinoma. The tongue of the CDDP and 5-MTP group showed that most of the tissue contained hyperkeratotic lesion and much less squamous cell carcinoma than the control group (**Figure 4C**). Immunohistochemical analysis revealed that the PCNA and p-STAT3 expression in the tongue tissue of the combination treatment group was less than those of the control and CDDP-treated groups (**Figure 4C**). This result indicated that the proliferation activity in the combination treatment group decreased more than that in the individual treatment groups.

## Discussion

A previous study reported that 5-MTP dose dependently reduces the growth of oral cancer cells, and the IC_50_ values of the OC3-I5 and OC3 cells are 400 and 1000 μM, respectively (16). Our results indicated that 5-MTP inhibited the growth of squamous cells, whereas the IC_50_ values of the SCC4 and SCC25 cells were 2.7 and 3.1 mM, respectively. These results demonstrate that the oral carcinoma cell lines were more sensitive to 5-MTP than the squamous carcinoma cell lines. Previous studies showed that 5-MTP reduces lung cancer A549 cell migration and invasion and attenuates A549 cancer growth by inhibiting COX-2 expression (20). Furthermore, fibroblasts release 5-MTP to reduce cell epithelial mesenchymal transition (EMT), migration, invasion, and metastasis by inhibiting p38 MAPK activation and NF-κB nuclear translocation (15). However, the mechanism of 5-MTP in squamous cancer had not been investigated. Our results showed that 5-MTP only and CDDP combined 5-MTP treatment reduced the proliferation of SCC25 cells by reducing Akt and STAT3 activation at 6-hour post treatment. The AKT/NF-κB signaling pathway has been indicated to be implicated in the development, progression, and metastasis of numerous cancers (21). Notably, 5-MTP and CDDP combined 5-MTP treatment suppressed STAT3 phosphorylation at 24-hour post treatment rather than Akt phosphorylation, which resulted in the reduction of SCC25 cell proliferation. These results may indicate that the inhibitory mechanism of the drugs is more relevant to STAT-3-miediated proliferation, which is independent of Akt signaling.

STAT3 plays an important role in many biological functions during the development of human tumor, including proliferation, survival, and inflammation (22). STAT3 acts as a transcription activator by binding to the promoter in a tyrosine phosphorylation-dependent manner, which is a critical modulator in EMT (23). In the present study, the combination treatment of CDDP and 5-MTP exerted a stronger reduction effect on the phospho-STAT3 level of SCC25 cells than the individual treatment. Thus, the STAT3 signaling pathway may be a critical mechanism involved in the reduction effect of 5-MTP and CDDP combination treatment on the growth of SSC25 cells. These results were associated with the possibility that 5-MTP and CDDP combination may be involved in invasion, migration, or metastasis. The effect of 5-MTP and CDDP combination on EMT in SCC25 cells should be investigated further.

STAT3 is an important target for developing effective immunotherapy (24). The inhibition of STAT3 activation in tumor upregulated the expression of several pro-inflammation chemokines and cytokines, which resulted in the infiltration of leukocyte into the tumor (25). The blockade of the constitutive expression of STAT3 in tumor reduced the expression of VEGF, IL-6, and IL-10, which neutralized the inhibitory effect of dendritic cell maturation (26). Furthermore, the STAT3^-/-^ mice showed that the anticancer cytotoxicity activity of natural killer cells and neutrophils increased (27). Therefore, STAT3 plays a role in the crosstalk between cancer and immune cells. The significant inhibition of STAT3 by 5-MTP or the combination of CDDP and 5-MTP in SCC25 cells should investigate the immunology regulation *in vitro* and *in vivo*.

HNSCC is usually induced in animal models by using 4NQO or 7,12-dimethylbenz(a)anthracene as a carcinogenic agent (28). Developed HNSCC animal models in several rodent species and solubility in drinking water are the advantages of 4NQO (29). Moreover, the molecular alterations induced by 4NQO in mouse mucosa mimic the human disease (28). In the present study, CDDP or CDDP combined with 5-MTP was administered to mice with 18-week 4NQO treatment for another 4 weeks, and the treatment successfully reduced tumorigenesis on the tongue tissue. With the same concentration of CDDP treatment, additional 5-MTP in the treatment enhanced the inhibitory effect on tumorigenesis. Taken together, our results indicate that the STAT3 signaling pathway is involved in the sensitized inhibitory effect of CDDP on HNSCC through the addition of 5-MTP *in vitro* and *in vivo*. CDDP is one of Food and Drug Administration–approved systemic therapies for concurrent use with radiation therapy for the treatment of HNSCC, which is the standard of care for non-operable locally advanced HNSCC (30). The effect of concurrent use of radiotherapy with CDDP and 5-MTP combination on HNSCC should be examined for developing more effective therapeutic strategy with less side effect.

## Materials and methods

### Ethics Statement and Experimental Animals

The animal experiments were performed in accordance with the Guide for Care and Use of Laboratory and approved by the Institutional Animal Care and Use Committee (IACUC, # 10908002) at the Scientific Integration Design Service Corporation (SIDSCO, Kaohsiung, Taiwan, ROC). The animals were cared for and raised under standard laboratory conditions in accordance with the Animal Use Protocol of SIDSCO. Six-week-old wild-type male Balb/c mice were purchased from BioLasco Taiwan Co. Ltd (Taipei City, Taiwan). All animal experiments were performed in specific pathogen-free environments in compliance with the guidelines of the Care and Use of Laboratory Animals. All mice were housed in a clean facility with standard laboratory conditions (12/12-h light/dark cycle, 22°C–24°C, 40%–60% humidity). All mice received human care and were given standard diet and water ad libitum. Mice were held for 14 days to acclimatize before the experiment.

### Cell Culture and Reagents

SCC25 and SCC4 cells were cultured in Dulbecco’s Modified Eagle Medium/Nutrient Mixture F-12 (DMEM/F12) containing 10% fetal bovine serum, 1% nonessential amino acids, and 1% antibiotic-antimycotic with 5% CO_2_ in a 37°C incubator. Cisplatin (CDDP), 5-MTP, and 4NQO were purchased from Sigma-Aldrich (Louis, MO, USA). All compounds were dissolved as stock solution and stored at −20°C.

### Cell Viability

SCC25 and SCC4 cells were seeded in 96-well culture plates at a density of 5 × 10^3^ per well. After overnight incubation, 5-MTP, cisplatin, or both was added to the cells at indicated concentrations. Cell viability was determined using CellTiter-Glo 2.0 Cell Viability Assay (Promega, Madison, WI, USA) in accordance with the manufacturer’s instruction. The results were detected by luminescence using a Varioskan™ LUX multimode microplate reader (Thermo Fisher Scientific, Waltham, MA, USA).

### Western Blotting

SCC25 and SCC4 cells were seeded in 6-wells at a density of 1.5 × 10^6^ per well. After overnight incubation, the cells were treated with CDDP, 5-MTP, or both at indicated concentrations for 24 h. The cells were washed with phosphate-buffered saline and lysed by adding ice-cold RIPA buffer supplemented with protease inhibitor and phosphatase inhibitor (Thermo Fisher Scientific, Waltham, MA, USA). The cell lysates were centrifuged, and the supernatant was separated in 10% sodium dodecyl sulfate-polyacrylamide gels and transferred onto PVDF membranes. The membranes were blocked with blocking reagent (Leadgene Biomedical, Taiwan), incubated with primary antibodies, and diluted with TBS containing 0.1% Tween 20 supplemented with 5% nonfat dried milk. The target proteins were probed with specific antibodies against p-STAT3 (ABclonal; 1:1000, AP0705), p-Akt (ABclonal; 1:1000, AP0637), p-p65 (Cell Signaling; 1:1000, 3033T), p-p38 (ABclonal; 1:1000, AP0526), PCNA (GeneTex; 1:3000, GTX100539), GAPDH (GeneTex; 1:10000, GTX100118). Enhanced chemiluminescence was used to detect the signals. The protein abundance of the samples was quantified using Image-J software following densitometric scanning.

### Evaluation of CDDP and 5-MTP Combination Treatment in 4NQO-Induced Oral Carcinogenesis

Six-week-old wild-type male Balb/c mice were fed daily with 4NQO (50 μg/mL) solution in their drinking water. After 18 weeks following the first dose of 4NQO, the mice were divided into three groups (six mouse each group). Group 1 received saline, Group 2 cisplatin (2 mg/kg), and Group 3 cisplatin (2 mg/kg) and 5-MTP (35 mg/kg). The dosage of CDDP and 5-MTP were followed the previous study (20, 31), and 5-MTP dosage was modified because it defined as supplement in present study. Cisplatin and 5-MTP were administered three times a week by intraperitoneal injection for 4 weeks. The Balb/c mice were sacrificed by carbon dioxide euthanasia. Tongue tissues were collected and fixed in 10% formalin for hematoxylin–eosin (H&E) and immunohistochemical (IHC) staining.

### Histopathology

Histopathological observations of the lung tissue were harvested were fixed in 10% formalin for 48 h. In brief, the tissues were embedded in paraffin and cut into 3 μm-thick section on slides and subjected to H&E and IHC staining to observe tumor proliferation and metastasis under a photomicroscope.

### IHC Staining

IHC staining was conducted using sections from paraffin-embedded blocks. In brief, 3 μm-thick sections sliced from paraffin-embedded specimens were prepared on slides for IHC staining following the manufacturer’s protocol of UltraVision Quanto Detection System HRP (Thermo Fisher Scientific Inc, Fremont, CA, UK). Sections were treated with hydrogen peroxide to block the endogenous peroxidase. The sections were incubated with primary PCNA (GeneTex; 1:500, GTX100539) and p-STAT3 (ABclonal; 1:100, AP0715) antibodies at 37°C for 1 h. The immunoreactions were visualized using the Epredia™ DAB Quanto Detection System (Fisher scientific, TA125QHDX) in accordance with the manufacture’s protocol.

### Statistical Analysis

Statistical analysis was performed with Prism 9.2 (GraphPad Software Inc., USA). Student’s t-test was applied to compare the statistical significance of difference between two groups. Statistical significance was considered at P < 0.05.

## Data Availability Statement

The original contributions presented in the study are included in the article/supplementary material. Further inquiries can be directed to the corresponding author.

## Ethics Statement

The animal experiments were performed in accordance with the Guide for Care and Use of Laboratory and approved by the Institutional Animal Care and Use Committee (IACUC, # 10908002) at the Scientific Integration Design Service Corporation (SIDSCO, Kaohsiung, Taiwan, ROC). The animals were cared for and raised under standard laboratory conditions in accordance with the Animal Use Protocol of SIDSCO. All animal experiments were performed in specific pathogen-free environments in compliance with the guidelines of the Care and Use of Laboratory Animals. All mice were housed in a clean facility with standard laboratory conditions (12/12-h light/dark cycle, 22°C–24°C, 40%–60% humidity). All mice received human care and were given a standard diet and water ad libitum. Mice were held for 14 days to acclimatize before the experiment.

## Author Contributions

Y-CS: study design, data collection and analysis, and preparation of the manuscript; C-CW: data collection and analysis, preparation of the manuscript; J-HW: data collection and analysis; S-AY: data collection; P-JC: data collection; T-ZH: data analysis; H-CC: study design and decision to publish. All authors contributed to the article and approved the submitted version.

## Funding

This work was supported in part by grants from Ministry of Science and Technology (MOST 109-2314-B-650-015), E-Da Hospital (EDAHP111015), and Kaohsiung Medical University Chung-Ho Memorial Hospital (KMHK-DK (整)109005~4).

## Conflict of Interest

The authors declare that the research was conducted in the absence of any commercial or financial relationships that could be construed as a potential conflict of interest.

The reviewer JT declared a shared affiliation, with no collaboration, with one of the authors, H-CC, and the reviewer LC declared a shared affiliation, with no collaboration, with several of the authors, Y-CS, C-CW, S-AY, T-ZH, to the handling editor at the time of the review.

## Publisher’s Note

All claims expressed in this article are solely those of the authors and do not necessarily represent those of their affiliated organizations, or those of the publisher, the editors and the reviewers. Any product that may be evaluated in this article, or claim that may be made by its manufacturer, is not guaranteed or endorsed by the publisher.
